# Tailoring of the Distribution of SERS-Active Silver Nanoparticles by Post-Deposition Low-Energy Ion Beam Irradiation

**DOI:** 10.3390/ma15217721

**Published:** 2022-11-02

**Authors:** Oleg Streletskiy, Ilya Zavidovskiy, Dmitry Yakubovsky, Natalia Doroshina, Alexander Syuy, Yury Lebedinskij, Andrey Markeev, Aleksey Arsenin, Valentyn Volkov, Sergey Novikov

**Affiliations:** 1 Department of Physics, M.V. Lomonosov Moscow State University, Leninskie Gory 1, 119991 Moscow, Russia; 2 Center for Photonics and 2D Materials, Moscow Institute of Physics and Technology, 141700 Dolgoprudny, Russia; 3 Institute of High Technologies and Advanced Materials, Far Eastern Federal University, 690091 Vladivostok, Russia

**Keywords:** silver nanoparticles, surface-enhanced Raman scattering, low-energy Ar^+^ irradiation, surface diffusion, resputtering, linear spectroscopy

## Abstract

The possibility of controlled scalable nanostructuring of surfaces by the formation of the plasmonic nanoparticles is very important for the development of sensors, solar cells, etc. In this work, the formation of the ensembles of silver nanoparticles on silicon and glass substrates by the magnetron deposition technique and the subsequent low-energy Ar^+^ ion irradiation was studied. The possibility of controlling the sizes, shapes and aerial density of the nanoparticles by the variation of the deposition and irradiation parameters was systematically investigated. Scanning electron microscopy studies of the samples deposited and irradiated in different conditions allowed for analysis of the morphological features of the nanoparticles and the distribution of their sizes and allowed for determination of the optimal parameters for the formation of the plasmonic-active structures. Additionally, the plasmonic properties of the resulting nanoparticles were characterized by means of linear spectroscopy and surface-enhanced Raman spectroscopy. Hereby, in this work, we demonstrate the possibility of the fabrication of silver nanoparticles with a widely varied range of average sizes and aerial density by means of a post-deposition ion irradiation technique to form nanostructured surfaces which can be applied in sensing technologies and surface-enhanced Raman spectroscopy (SERS).

## 1. Introduction

The controllable nanostructuring of surfaces by the formation of the nanoparticles of noble metals such as gold, silver and copper, which support the excitation of localized surface plasmon resonance (LSPR), are of increasing interest for a wide range of applications, such as non-linear optics, electronics, catalysis, sensing, enhanced hot-electron and photocurrent generation [1,2,3,4]. Controlled and reliable field enhancement (FE) effects associated with the excitation of plasmons in resonant metal nanostructures [5,6,7] are a potential key to the development of new sensors, and other practical applications of the surface-enhanced Raman spectroscopy (SERS) [1,8,9,10,11]. Silver is one of the metals with excellent plasmonic properties that can be used in the abovementioned fields [12,13,14]. Additionally, the biomedical properties of silver and silver-based structures are of recent interest [15,16,17]. There are many strategies for the fabrication and nanostructuring of surfaces by plasmonic structures involving silver. Various approaches to the plasmonic-active silver formation, such as deposition by evaporation or sputtering, high-fluency ion implantation, chemical- and photoreduction, colloidal solutions condensation, electron beam lithography, etc., have been suggested [18,19,20,21,22]. Many of them grant an attractive efficiency of the nanomaterial formation. The important aspects, often limiting the practical application of some listed above methods are the cost-effectiveness of manufacturing, the simplicity of the fabrication procedures and, on the other hand, the necessity of the reproducibility and large-area homogeneity of the resulting structures.

Therefore, the methods of the formation of nanostructured surfaces via several-step processes are being extensively analyzed and developed [23]. A number of proposed approaches are aimed at the “top-down” post-deposition treatment of Ag films rather than at the formation of the nanoparticles in the course of the deposition. Silver films may be irradiated by high-energy ion beams [21,24,25] or undergo high-temperature annealing [26], thus resulting in Ag nanostructuring. Middle- and low-energy ion irradiation also allows the development of the surface of a silver film and the formation of separate nanoparticles [27,28]. Low-energy techniques are favorable for the formation of the structures on various substrates, owing to the reduction of the substrate heating during the synthesis, allowing the modification of the structures deposited on the substrates vulnerable to heat, such as polymers and fabrics. The modification of the plasmonic structures via low-energy ion irradiation is of recent interest [29,30,31], as this technique allows altering the structure without the amorphization or ion-beam mixing [28].

The aim of the study was to prove that low-energy ion irradiation of Ag films makes it possible to manufacture Ag NPs with controllable properties which can be applied as SERS-active substrates. Additionally, the ion-induced processes were investigated. As point-of-care diagnostics via SERS for biomedical, biochemical and food analysis are widely developed nowadays [32,33], it was of our particular interest to find out which parameters ensure the ion-beam treatment with low substrate damage. To ensure a variable effect on the substrate and silver film, we have irradiated Ag films of various thicknesses with various doses of low-energy (150 eV) Ar^+^ ions.

Previously, Ag film treatment by plasma exposure was reported in [28,34,35]. Among these works, the Ar^+^ flux adjustment and its effect on the resulting silver nanostructures were studied in [34], where variable bias was applied to the substrate, allowing for the control of the morphology of the Ag NPs. However, bias enlargement generally leads not only to the increase of the particle energy but to the increase of ion flux as well [36], impeding the independent control of the ion dose and energy during the plasma exposure. In our study, we demonstrate that the Ar^+^ beam created by the Hall-effect ion source may be applied to irradiate the Ag nanostructures, allowing for simple and reliable variation of the dose of Ar^+^ with selected energy. In this work, we discussed the impact of the magnetron deposition time and ion-beam irradiation dose on the morphological properties of the silver nanoparticles, which allowed us to summarize the influence of ion-induced sputtering, surface diffusion and defect-assisted nucleation on the properties of ion-beam-treated Ag nanostructures. We showed that the discussed method ensures the control of the size and surface coverage of Ag NPs, and we anticipate the obtained results to be very promising for their further plasmonic sensing applications.

## 2. Materials and Methods

### 2.1. Fabrication Process

The coating process was carried out in a high-vacuum chamber with a preliminary pumping up to 1.3 × 10^−3^ Pa, followed by synthesis in an argon atmosphere at the pressure of 1 × 10^−1^ Pa. The diameter of the used 99.999% Ag target was 75 mm, while the distance between the substrate and the target was 200 mm. The angle between the flux of the Ag particles and the substrate surface was kept at 45°. During the deposition, the samples were fixed on the substrate holder, located on the carousel of the 20 mm diameter with five positions for substrate holders. Four of them were shuttered from the flux of the particles during the deposition. The active substrate holder was exposed for the particle beam. Prior to the deposition, the surface of the target was decontaminated by 60 s magnetron sputtering at the discharge power of ~30 W, during which the flux of the sputtered particles was aimed at the substrate holder which had no loaded samples. The cover glass Deltalab D101818 and silicon wafer were used as substrates. The structures deposited on glass substrates were used for transmittance measurements, which allowed us to investigate the absorbance spectra. Before applying the coating, the substrate surfaces were etched by the ion beam of 1 keV energy for 5 min. A thin coating was deposited by radio-frequency magnetron sputtering of a silver target. The discharge power was approximately 30 W. Three sets of samples, indicated as “I”, “II” and “III” throughout the manuscript, were deposited within 22, 45 and 67 s, respectively. The thicknesses were 10, 20 and 30 nm, respectively. The thickness of the films was measured by the detector based on a quartz oscillator (Inficon Corp., Moscow, Russia). The sensor worked in the mass assessment mode. The value of the mass of the deposited material detected after the deposition of each sample was used to estimate the thickness of the films. After the deposition, the argon ion irradiation of 150 eV energy and 20 mA/cm^2^ current density was applied to the deposited films. The angle between the beam and the substrate surface was 45°. The samples were irradiated at the doses of 2.2 × 10^16^ ion/cm^2^, 4.5 × 10^16^ ion/cm^2^ and 6.7 × 10^16^ ion/cm^2^. The ion beam was produced by the ion source with cold hollow cathode Klan 53-M (Platar Corp., Moscow, Russia).

### 2.2. Sample Characterization

Samples visualization was implemented by scanning electron microscopy (JEOL JSM-7001F, Tokyo, Japan), transmission electron microscopy (JEOL JEM-2100, Tokyo, Japan) and by an optical microscope (Nikon LV150, Tokyo, Japan) with a digital camera DS-Fi3. UV–visible absorption (UV–Vis) spectra of the samples were measured by the UV–Vis–NIR spectrophotometer Agilent Technologies Cary 5000 (175–3300 nm, Santa Clara, CA, USA).

Transmission electron microscopy (JEOL JEM-2100, Tokyo, Japan) was used to characterize the nanoparticles. The used electron microscopy grids consisted of copper frames with 40 μm cells and thin (~5 nm) polymer film deposited on the upper side of the frame. To transfer the structure to these grids, the samples were mechanically pressed to the polymer-coated side of the grids and removed after 5–10 s of grid-sample interaction.

The chemical state of the samples was controlled by X-ray photoelectron spectroscopy (XPS). The measurements were performed by the Theta Probe (Thermo Scientific, Walthamm, MA, USA) system with a monochromatic Al Ka (1486.6 eV) X-ray source. The photoelectron spectra were acquired in the fixed analyzer transmission mode with the pass energy of 50 eV. The cleaning of the surface by Ar ions was not carried out, since ion etching could cause a change in the chemical state of Ag NPs. The SERS performance of the silver nanoparticles was investigated by the Raman microscope Horiba LabRAM HR Evolution (Horiba, Kyoto, Japan). The measurements were performed with a 632.8 nm laser wavelength, 600 lines/mm diffraction grating, 0.5 sec. integration time, ×100 objective (N.A. = 0.90), and with an incident power ~0.3 mW. The spot size was ~0.43 μm, scanned area was 10 × 10 μm^2^, and scanning step was ~0.7 μm (15 × 15 points in map).

Before SERS measuring, the samples were covered with the 10^−7^ M solution of crystal violet (CV) in distilled water and dried under ambient conditions.

## 3. Results and Discussion

**Figure 1:**
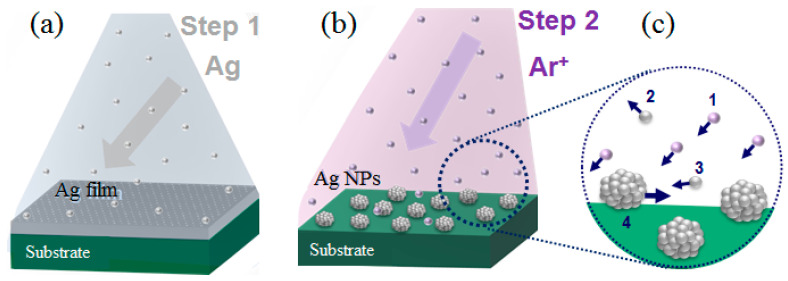
The schematic illustration of Ag NPs formation. (**a**) Magnetron deposition of the silver films. Film irradiation by low-energy low-dose Ar^+^ ions (**b**) leading to the formation of the nanoparticles. Characteristic processes (**c**): 1—Ar^+^ flux; 2—Ag sputtering; 3—Ag resputtering; 4—surface diffusion of the adatoms and adsorbed clusters enhanced by ion irradiation.

The low-energy ion irradiation of the Ag films leads to their structural rearrangement. The film modification can be controlled by a dose variation, inducing processes such as sputtering and resputtering of the material and the initiation of the surface diffusion of adatoms and clusters (Figure 1).

The thickness of the deposited silver film is another parameter that determines the structure of the resulting material by affecting the degree of the interaction between ion beam and substrate. Substrate exposure to a low-energetic ion beam initiates multi-step structural rearrangement of irradiated material which involves the accumulation of the defects and subsequent relaxation resulting in the formation of point defects on the surface [37]. The defects act as favorable nucleation centers of the metal particles [36,38,39]. Such particles are usually smaller than the ones affected by the diffusion and coalescence, and their size varies slightly in different conditions: e.g., in [39] the silver particles “pinned down by defects” were capped at 5 nm size, while in [40] we have found out that the ion-induced defect formation enhances the number of sub-12 nm Ag particles. In order to carry out the systematic study of the influence of the aforementioned parameters on the modification of silver film and the possibility of controlling the size and surface coverage of formed nanoparticles, samples I, II and III (see Section 2.2 for details) were analyzed. The samples were irradiated with three various doses and with the fixed energy of 150 eV Ar^+^ ions. The Ar^+^ energy of 150 eV was chosen according to the studies, indicating that energies exceeding 200 eV can lead to ion-beam mixing, ion incorporation and silver amorphization [41,42,43,44].

### 3.1. XPS Analysis

**Figure 2:**
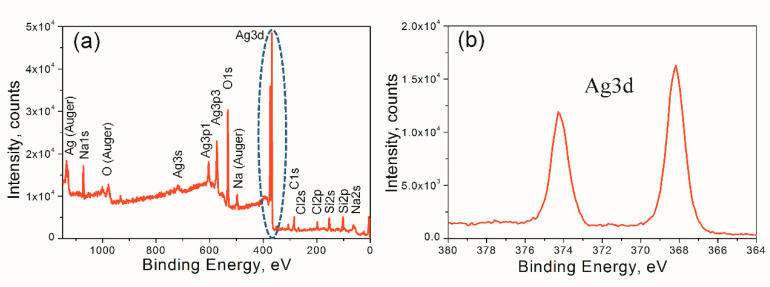
(**a**) XPS spectra of one sample of Ag NPs and (**b**) close-up image of Ag3d peaks.

In order to clarify that the used method allows the formation of Ag NPs without significant impurities, XPS analysis of sample II, which was irradiated with a 4.5 × 10^16^ ion/cm^2^ dose deposited on a Si substrate, was performed (Figure 2a,b). The Ag3d line reveals the doublet Ag3d_5/2_-Ag3d_3/2_ with the peaks at 368 and 374 eV. They can be fitted by two Gaussian lines with the full width at half maximum (FWHM) of 0.85 eV, corresponding to the resolution of the XPS set-up. It confirms that the formed NPs consist of silver in a metallic state [45]. On the other hand, the lower energies of 367.2–367.8 eV corresponding to the Ag(I)/Ag(III) state of oxidized silver atoms [46,47] were not observed in the spectrum. The ratio of the intensity of O1s to the intensity of Si2s and Si2p lines normalized to the relative sensitivity factors of corresponding lines is close to 2:1, which confirms that the oxygen detected by XPS was observed primarily in the SiO_2_ substrate. The concentration of the detected carbon is low, and its presence apparently corresponds to the minor surface carbon contamination due to the interaction of the samples with the atmosphere.

### 3.2. TEM Analysis

**Figure 3:**
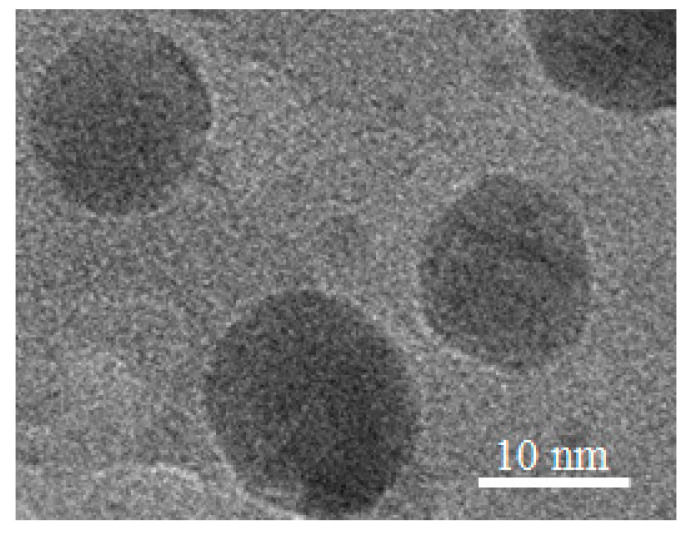
TEM images of NPs with sizes near 10 nm formed by ion-beam irradiation.

The TEM images (Figure 3) show the NPs of about 10 nm in size formed by ion-beam irradiation of sample II with a 2.2 × 10^16^ ion/cm^2^ dose deposited on the Si substrate. This result confirms that ion irradiation leads to the formation of individual NPs from continuous films. As the SERS intensity of continuous films is lower than the one of individual nanoparticles [39,48], the tunable top-down two-step approach which allows for the formation of separate plasmonic-active NPs from the Ag films may be of interest for the SERS applications. The important aspect of the plasmonic performance is the distribution of the particles affecting the presence of the hotspots, which depends both on the size of the particles and the lengths of the gaps in between [49,50]. However, as the nanoparticle transfer from the substrate to the TEM grid by mechanical pressing does not guarantee that the distribution of the deposited particles is preserved on the grid, rigorous analysis of the samples’ morphology was carried out by SEM.

### 3.3. SEM Analysis

**Figure 4:**
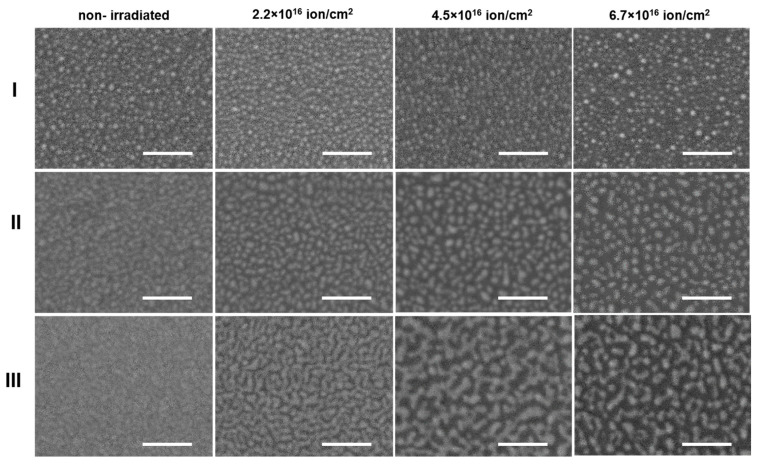
SEM images of Ag nanoparticles with different initial thicknesses: I-10 nm, II-20 nm and III-30 nm (shown in rows) irradiated by ion doses indicated on the top of each column. The scale bar indicates 100 nm length.

In Figure 4 one can observe the SEM images of the NPs and coatings deposited on the Si substrates. The samples were prepared within various magnetron sputtering periods and irradiated with various doses of ions. The alternation of the parameters resulted in a significant change in the films’ morphology. The increase of the deposition time results in the fading of the images of non-irradiated samples, which apparently indicates that their roughness decreases with the increase in the thickness of the deposited coating. The ion beam treatment of the samples induces their nanotexturing, leading to the formation of the individual Ag nanoparticles, with their size and aerial density depending on the ion dose. For sample III, the applied irradiation doses have not induced the complete separation of the nanoparticles, as the branched structures were observed for treated sample III.

**Figure 5:**
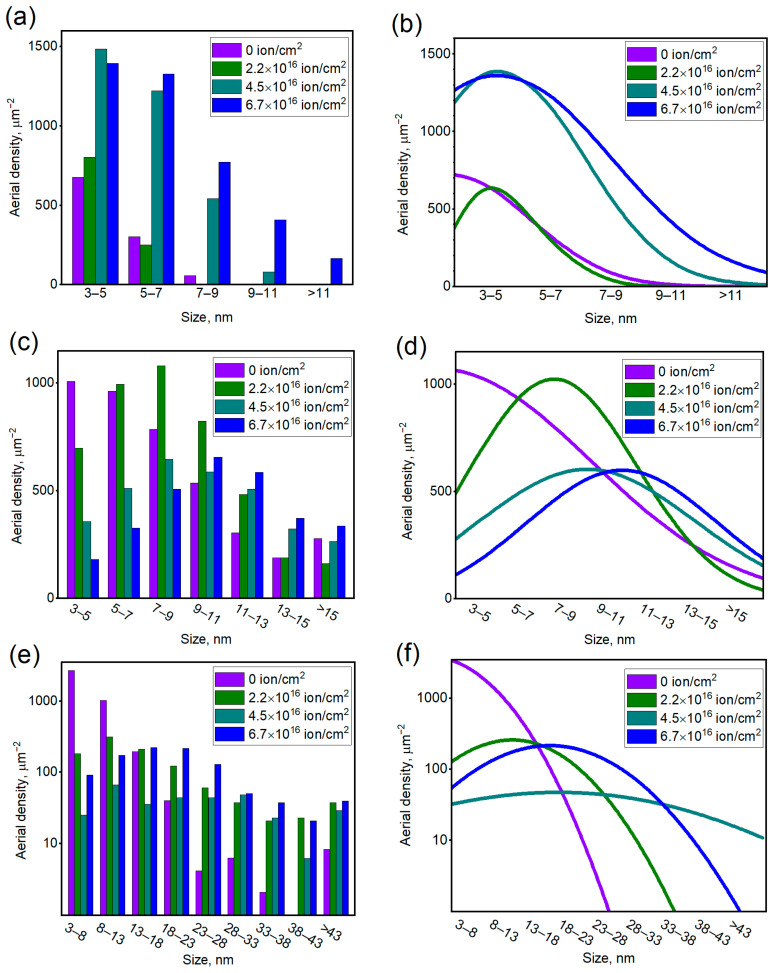
NP size distribution for sets I (**a**), II (**c**) and III (**e**) irradiated with various doses; fittings of the distributions with Gaussian lines for sets I (**b**), II (**d**) and III (**f**).

To investigate the evolution of the samples, SEM images were processed with Gwyddion software (Gwyddion 2.61, open-source software, Brno, Czech Republic, http://gwyddion.net/, accessed on 25 August 2022). The NP size estimated via this software is a doubled radius of the disc of the projected area similar to the one of the NP (equivalent disk radius). Figure 5a,c,e shows the size distribution of the heterogeneities in Ag film thickness (nanoparticles and convexes) observed on the surface of the samples I, II and III, while the Gaussian fittings of the distributions are shown in Figure 5b,d,f). The detected features of the size close to SEM resolution were not taken into consideration. The average sizes of the morphological features of non-irradiated samples I, II and III were 4.7 nm, 8.1 nm and 7.3 nm, respectively.

**Figure 6:**
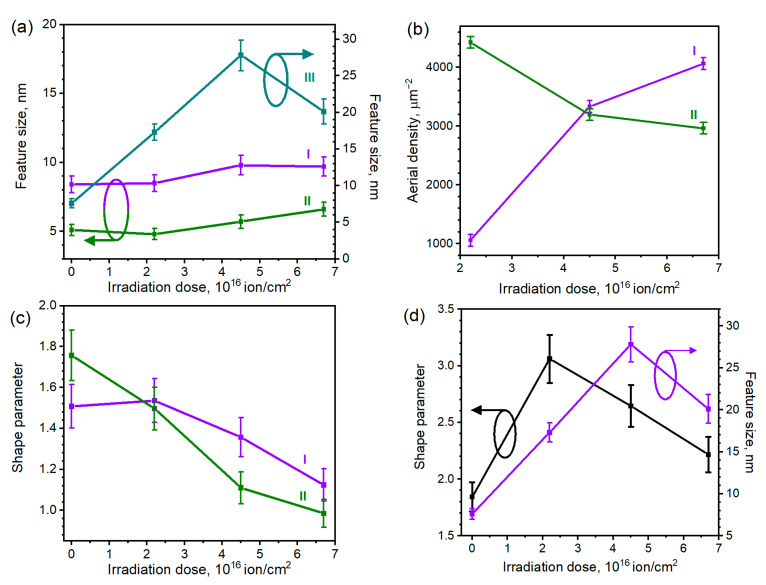
(**a**) Average size of the particles and convexes of the samples irradiated with various ion doses. (**b**) The dependence of average aerial density on the ion irradiation dose for irradiated sets I and II. (**c**) The dependence of the shape parameter of the nanoparticles on the ion irradiation dose for sets I and II. (**d**) The dependence of the shape parameter and sizes of characteristic morphological features on the ion irradiation dose for set III.

Size distributions of the NPs obtained after ion irradiation of sample I (initial thickness 10 nm) and their approximations with Gaussian lines are shown in Figure 5a,b. The size of the NP enlarged with the irradiation dose from 4.3–4.7 nm (for non-irradiated and 2.2 × 10^16^ ion/cm^2^-irradiated samples) to 5.5–6.3 nm for the samples irradiated with the (4.5–6.7) × 10^16^ ion/cm^2^ dose are shown by the violet line in Figure 6a.

The distributions of the particles observed for irradiated sample II (initial thickness 20 nm) (Figure 5c) and their Gaussian fittings (Figure 5d) differ significantly from the ones of irradiated sample I. The average size of the NPs observed for the sample irradiated by 2.2 × 10^16^ ion/cm^2^ dose is 8.4 nm. The increased ion treatment dose resulted in the decrease in aerial density of the particles and their enlargement. As shown by the green line in Figure 6a, for the 4.5 × 10^16^ ion/cm^2^-irradiated NP, the average size was 9.6 nm, while for the 6.7 × 10^16^ ion/cm^2^ treatment, it reached the value of 10.5 nm.

For set III (initial thickness 30 nm), the resulting agglomerates were, apparently, not entirely separated from the continuous Ag layer by ion irradiation. Therefore, the morphological features of this sample are hereinafter referred to as “convexes”. The ion irradiation of sample III resulted in the surface development and the formation of these agglomerates consisting of convexes interconnected with narrow “bridges”. The size distributions (Figure 5e) in this case are significantly wider than for the previous samples. Agglomerates size enlargement and distribution widening with the increase in the dose was observed for sample III. Such a trend resembles the one observed for separate NPs of sample II. However, for sample III, the largest morphological features of the lowest surface density were observed at the average irradiation dose of 4.5 × 10^16^ ion/cm^2^. Such irradiation parameters are favorable for the formation of mostly laced agglomerates, whereas a subsequent dose increase to 6.7 × 10^16^ ion/cm^2^ leads to the separation of the particles. We should note that morphologically, set III features irregular shapes, so the depicted “size” can poorly reflect the real width, extension, or any linear dimension of the branched particle. The Gaussian fittings (Figure 5e) of the irradiated sample III also poorly reflect the distributions, clearly showing only the tendency of the widening of convex distribution with the introduction of the ion irradiation.

### 3.4. Discussion of the Ion-Induced Processes

The differences in the distribution of the Ag NPs formed from the films with various thicknesses can be explained by various manifestations of the irradiation-induced processes, such as the sputtering and the resputtering of the material; the enhancement of the mobility of the resputtered adatoms and nuclei; and the defect-induced nucleation of the resputtered material. As the initial thickness of sample I (~10 nm) is low, the morphological features of set I may be related to the prominent ion-substrate interaction. The high aerial density of the sub-7 nm particles in this case is explained by the set of interconnected processes: first, during the ion-beam treatment, the surface of the substrate was exposed and irradiated, resulting in the formation of the defects on it. Second, the defects formed on the substrate surface act as favorable nucleation centers for the surface-diffusing silver adatoms and clusters formed during ion irradiation [38]. In the studied case, the particles sized below 7 nm were observed for all of the samples, which confirms that their formation may be attributed to the nucleation of ion-induced defects. Notably, the value of 7 nm lies in the range of the previously observed values of the capping of defect-induced nanoparticles (5 nm in [39], 12 nm in [40]), which confirms the defect-induced origin of the sub-7 nm particles. As for the “tail” of the distribution, i.e., particles exceeding 7 nm, they were enlarged and widened with the irradiation dose increase. This observation may indicate that the increase in the ion irradiation dose leads to the enhancement of the mobility of the resputtered adatoms, and consequently amplifies the rate of adatom capture by growing islands on the substrate surface, resulting in nanoparticle enlargement [51]. This effect apparently causes the enlargement of the NPs with the increase in the irradiation dose.

For set II, the ion-induced defect formation on the substrate surface for thicker films is suppressed. In this case, the number of nucleation centers on the substrate is decreased, and ion irradiation predominantly affects nanoparticles by enhancing their surface mobility, thereby leading to the enlargement of the particles and a decrease in their numbers. The overall aerial density of the particles for samples I and II irradiated with various doses is shown in Figure 6b. The results for set III are not shown, as in this case, the Ag agglomerates were not entirely separated from the continuous Ag layer. The enlargement in the particle number with the dose increase observed for set I confirms the significant role of ion-induced defect formation in the distribution of the nanoparticles, due to the creation of more nucleation centers with the increase in the dose. In turn, the decreasing trend of the aerial density for set II may prove that particle condensation on the defects affects thicker samples to a much lesser extent, whereas the observed enlargement of the particles with the irradiation is due to the resputtering and coalescence.

For sample III, the results showing the enlargement of the particles and branching of the structure are in correspondence with [52] findings illustrating the evolution from an island Ag film to a “wormhole” network with the increase in Ag flux condensing on the substrate. As shown in [52], the irregular nanoparticle formation originates from the surface diffusion of adsorbed atoms and particles leading to their coalescence. However, large morphological features are unlikely to be mobilized by the ion beam, therefore the branching of the structure is induced by the diffusion of the resputtered adatoms and clusters. This process results in the condensation of the resputtered material on the edges of the stable particles leading to the formation of “bridges”.

The variation in the average feature size with the irradiation dose (Figure 6a) confirms the effect of the NPs’ size increase with a prolonged ion-beam treatment. The analysis of the processes taking place during the treatment shows that low-energy ion beam irradiation of the silver surface results not only in silver sputtering leading to NP formation from a continuous film but also in Ag resputtering and surface diffusion enhancement which caused Ag islands enlargement and the widening of their size distribution with prolonged irradiation.

To investigate the morphological changes of the sets, the shape parameter of the particles was assessed as an (*R_c_* − *R_o_*)/*R_o_*, where *R_c_* and *R_o_* are the radii of the circumscribed and inscribed circles [53]. Thus, the shape parameter of the round particle is 0, while its enlargement indicates the elongation of the particle. For the studied Ag NPs, the radii were assessed for the particles over 3 nm in size via the Gwyddion software.

In Figure 6c, the averaged shape parameters of sets I (purple line) and II (green line) are shown. The analysis of both sets revealed the decreasing trend of the shape parameter, indicating the particles become more spherical with the dose increase (as the sphericity cannot be revealed by the SEM images, we discussed the analysis of the NPs shape below). In our understanding, the sphericalization of the NPs is related to the effects of the texturing of the silver under ion beam irradiation [54]. In [54], it is shown that various grains of polycrystalline Ag films are sputtered to various degrees depending on the manifestation of the ion channeling effect. However, unlike the results reported in [54], this effect does not lead to the roughening of the continuous film but results in the preferential sputtering of the crystallites oriented unfavorably for channeling. While the resputtering takes place, the size and the number of the unfavorably-oriented crystallites and amorphous fragments apparently diminishes, whereas the transfer of the resputtered material nourishes the favorably-oriented crystallites. As the particles become more single-crystalline, the shape of the NPs becomes more spherical. The monocrystallinity of the NPs is an important aspect of their plasmonic performance, as it drastically affects the speed of their tarnishing [19]. Therefore, the analysis of the SERS stability and crystallinity of the Ag NPs manufactured by the studied method will be of interest for our further studies.

The averaged shape parameter of sample III irradiated with various doses (Figure 6d, black line) differs significantly from the ones of samples I and II. The shape parameter value of the samples irradiated with (2.2 − 4.5) × 10^16^ ion/cm^2^ dose considerably exceeds the ones of the non-irradiated sample and the sample irradiated with the dose of 6.7 × 10^16^ ion/cm^2^. This tendency to some extent resembles the variation of the average size of the morphological features of set III with the dose (Figure 6d, purple line). We suggest these tendencies are caused by similar processes, with them being governed by the surface diffusion of the resputtered material at the dose range of (0 − 4.5) × 10^16^ ion/cm^2^ while being predominantly affected by the preferential sputtering of the unfavorably-oriented and disordered clusters discussed for sets I and II at the (4.5 − 6.7) × 10^16^ ion/cm^2^ range. The elongation and the branching of the particles are interconnected in this case, as both of the processes are caused by the formation of the narrow “bridges” between the individual particles resulting in the formation of the “wormhole” network rather than the enlargement of the nanoparticles. The initial increase in the shape parameter of set III with the dose enlargement is notably different from the decreasing tendency observed for samples I and II. As set III is apparently formed by the convexes on the continuous film rather than by the NPs, the ion-induced surface diffusion of the resputtered material is more prominent for this set. Intensive diffusion leads to the condensation of the “bridges”, while the “erosion” of the material results in the decrease of its overall thickness rather than in the shrinking of the NPs. Apparently, both of the decreasing trends observed in Figure 6d for larger doses indicate the depletion of the continuous Ag layer, which results in the sputtering of the unfavorably-oriented fragments of the individual NPs rather than the overall “erosion” of the film.

The observed formation of “bridges” proves that the ion-induced diffusion affects mostly the resputtered adatoms and clusters, while the nanoparticles are mostly immobile. Therefore, the low-energy ion irradiation technique may be applied to form the SERS-active structures on the vulnerable analytes (organic films, etc.), as the structure in the vicinity of the enhancing agent will remain shadowed from the ion beam.

To discuss the possibility of the formation of the plasmonic-active media on the surface of various samples, the variability of the Ag NPs formation on different substrates should be addressed. We haven’t observed any influence of the surface morphology on the Ag NPs distribution, as the ion irradiation of ~200 eV at similar ion currents is known to create ~50–100 nm-sized ripples on the surface [55], leading to the non-uniform distribution of the nanoparticles on the surface [28]. In our case, as no non-uniform distribution was observed, we suggest that ion-induced ripple formation does not affect the nanoparticle distribution. Nevertheless, there are several additional factors depending on the substrate type, that may affect the distribution of the nanoparticles.

Firstly, the processes of the formation of ion-induced defects and surface diffusion may occur differently on various substrates. The materials with ionic bonding are more likely to undergo modification under ion irradiation than the materials with covalent bonding, as the preferential sputtering leads to the removal of the anions from the subsurface layers, resulting in the pronounced formation of point defects [56]. Therefore, we suggest that the aerial density of the small (≤7 nm) particles, originating from the condensation of the resputtered material on defects, will be higher for the structures deposited on the materials with ionic bonding than for the ones deposited on the materials with covalent bonding, such as Si or SiO_2_.

Secondly, the wettability between the Ag NP and the substrate affects the shape of the condensing particles [57]. Generally, the shape of the nanoparticles can be estimated via the capillary model of heterogeneous nucleation [58]. According to this model, the wetting angle depends on the relation between the surface energies of the substrate and nanoparticle, contact surface and thermodynamical factors. In our case, the surface energy of silver nanoparticles (~1100 mJ/m^2^) [59] is significantly larger than the one of silicon and glass (~100 mJ/m^2^) [60,61]. As a result, the condensation of the particles differs slightly from the homogeneous nucleation process (i.e., from the condensation taking place in supersaturated vapor without contacting a solid surface), which means that the Ag nanoparticles condensed on silicon and glass substrates have spheroidal or ellipsoidal shapes. As for some other substrates, the poor wettability of silver (i.e., spheroidal or ellipsoidal shape of nanoparticles) is generally expected on the oxide substrates, while the wetting behavior of condensing silver is observed on Al, Au, Cr, Cu, Ge, Ni, Sn, Ti, leading to the formation of ultrathin Ag layers on these surfaces [62].

Something this is noteworthy is that ion assistance may introduce some additional effects into the nucleation process. Ion-induced surface development may change the contact surface, thus reducing the critical nuclei size [58], while particle charging may lead to the “self-repulsion” of the surface charges, thus facilitating particle sphericalization [63]. Additionally, the formation of charged particles can take place both during the ion-induced modification and the magnetron deposition, as the conventional magnetron sputtering technique provides the up-to-10%-ionized flux [64]. However, it is unclear if the charge will retain in the process of particle–substrate interaction during the condensation, therefore this aspect remains a subject of further studies. In turn, the modification of the surface is unlikely to affect the wettability, as the nanoparticles are mostly immobile during the ion beam exposure. Therefore, the substrate located in the vicinity of the nanoparticle is shadowed from the ion beam, so the modification of the contact surface is unlikely.

Thirdly, the substrate type may also affect the mobility of the adatoms. As the variation of the substrate material changes the activation energies of diffusion and desorption [65,66], the substrate may affect the processes of ion-induced resputtering and coalescence of the adatoms. The process of the diffusion and condensation of the two-dimensional adatom gas depends on the morphology and texturing of the surface, as well as on the chemical interaction between the surface and adatoms [67,68]. Additionally, the ion beam activity not only induces surface diffusion but also leads to the trapping of incident particles, the sputtering of the substrate atoms and the amorphization of the surface [69], which may affect the adatom mobility as well. To our knowledge, no systematic study on the silver surface diffusion on silicon and glass substrates was reported, as well as on the influence of ion-beam-affected processes on the material condensing on these substrates. Thus, we can only use the simplified approach to estimate the substrate effect on adatom mobility. It shows that the energy of the diffusion activation of Ag on Si(111) is 0.33 eV [70], while for Ag on amorphous SiO_2_ it is 0.32 eV [71]. Therefore, we do not expect any significant differences in the surface diffusion of adatoms on the used substrates; however, further studies on the irradiation-related variation of the diffusion process may reveal the difference.

To sum up, the difference in the NP particle size distribution on different substrates may in general be caused by the variation of the morphology of substrates, the variability of the processes of condensation, surface diffusion and wetting. However, we do not expect a significant difference in the processes of condensation, surface diffusion of resputtered material as well in the wetting. As the wettability of Si and glass to Ag is poor, we expect a spheroidal or ellipsoidal shape of Ag NPs in the studied case.

The important practical aspect of the presented analysis of the Ag NPs’ distributions is the demonstration of various exposure of the coated substrates to ion irradiation. The size distribution in the NPs of set II shows that the ion-induced defect formation is less prominent than the one in set I. It allows us to suggest that for set II, the processes related to the interaction between the substrate and ion beam are suppressed by the thicker Ag layer. This is confirmed by the particle increase and the distribution widening tendency with irradiation dose increase, which may be adequately explained by the ion and silver interaction summarized in Figure 1c. Therefore, the analysis of the plasmonic properties of this set is of interest, as the initial thickness of sample II makes it possible to deposit the Ag NPs on the surface of various samples, damaging them only slightly, which is beneficial for their analysis. As for sample III, it is known that the SERS performance of the continuous films is lower than the one of the individual nanoparticles [39,48]. We should also note that for set III, ion irradiation induces the increase in the distance between the convexes, which deteriorates the SERS amplification of the set. Thus, set II was chosen for the subsequent plasmonic studies. The hotspot-based approach [49] allows us to assume that both high aerial density (i.e., low distance between particles) and large particle size are beneficial for the SERS performance of the samples. As shown in Figure 6a,b, the ion irradiation of sample II led both to the enlargement of the particles and the reduction of their aerial density. Thus, the increase in the irradiation dose affects the SPR-related characteristics of the samples both in negative and positive ways. This determined our choice of the sample irradiated with a moderate dose of 4.5 × 10^16^ ion/cm^2^ for subsequent SERS-related studies discussed in Section 3.5. However, the relation between the plasmonic performance of the samples and their deposition conditions requires further studies.

### 3.5. UV–Vis Spectroscopy and SERS Studies

**Figure 7:**
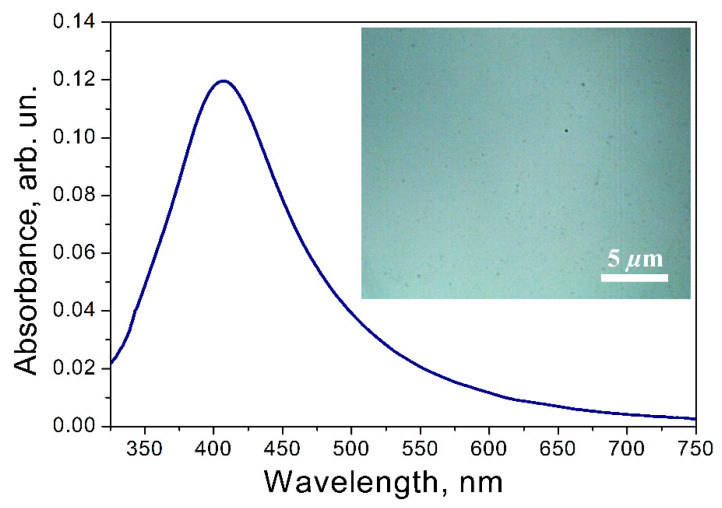
Extinction spectrum from the Ag NPs of sample II irradiated by a dose of 4.5 × 10^16^. The insert is an optical image.

The selected sample was also characterized by linear and Raman spectroscopy. The experimental extinction spectrum of Ag nanoparticles on the glass substrate recorded for sample II and the 4.5 × 10^16^ dose are shown in Figure 7. The LSPR band with a maximum at near 418 nm was observed in the UV–vis absorption spectrum of the sample.

**Figure 8:**
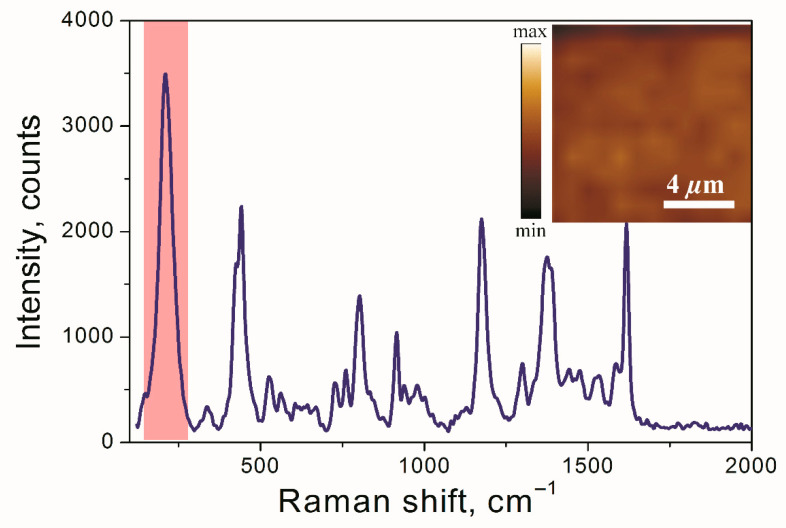
SERS spectra of CV with the concentration of 10^−7^ M adsorbed on ensembles of silver NPs. The shaded column marks the range used for the SERS mapping, which is shown in the insert.

To estimate the applicability of the fabricated samples for SERS, the studies of Raman spectroscopy were carried out. The spectra of the crystal violet solution of the 10^−7^ M concentration are shown in Figure 8.

The optical images and SERS mappings (inserts of Figure 7 and Figure 8) demonstrate a homogeneous distribution of Ag NPs on the surface and a low spatial variation of the intensity of the SERS signal consequently. To compare the SERS signal intensity with the intensity of the Raman signal of CV on a SERS-inactive substrate keeping the same experimental parameters, the analytical enhancement factor (EF) was used [72]. The average EF was estimated to be ~0.82 × 10^5^ for the ensembles of NPs. Notably, the used Raman excitation wavelength of 632 nm is relatively far from the observed SPR peak of 418 nm, which demonstrates the versatility of the properties of the samples. We suggest that the use of the 488 nm excitation wavelength can enhance several times the EF of the studied nanoparticles [73]. Howbeit, the observed EF value lies in the range of the hotspot-affected SERS of Ag island films and substantially exceeds the value of 10^3^ typical for the Ag nanoparticles unable to form the hotspots [74]. This confirms our previous assumptions regarding the nanoparticle distribution effect on the manifestation of the hotspot effect (Section 3.4). In addition, the reported results pave the way for subsequent studies, which are to be aimed at the evaluation and tuning of the contribution of the hotspots to the plasmonic performance of the Ag NPs tailored by low-energy ion irradiation.

## 4. Conclusions

The Ag nanoparticles manufactured by the magnetron deposition of Ag film and its subsequent low-energy ion beam irradiation were studied in the current work. The possibility of the controlled nanostructuring of the surfaces with Ag NPs by altering the film thickness and irradiation doses was demonstrated. The SEM analysis shows that ion irradiation typically results in an increase in the average size of silver nanoparticles as well as the broadening of their distribution. These processes are due to the combination of irradiation-induced sputtering, resputtering and the enhancement of the surface mobility of adatoms and nuclei. Higher surface exposure to the ion beam was observed for the 10 nm-thick sample. In this case, the interaction between the ion beam and the substrate leads to the formation of the defects—favorable nucleation centers of sub-7 nm Ag NPs. However, branched NP agglomerates were observed for the 30 nm-thick sample, indicating that applied irradiation doses do not result in the NPs’ separation for thicker Ag films. In turn, the formation of the defects on the substrate of set II (the thickness of the initial film is 20 nm) is suppressed, compared with set I (the initial thickness is 10 nm). The listed factors allowed us to choose the irradiated 20 nm-thick Ag film for the SERS studies. The formed Ag NPs manufactured from the film of 20 nm in thickness with the 4.5 × 10^16^ ion/cm^2^ Ar^+^ dose were also characterized by linear spectroscopy and SERS. Such particles showed excellent plasmonic properties, indicating that ion irradiation is a promising way for the formation of the structures for SERS applications.

## Data Availability

The data that support the findings of this study are available from the corresponding author upon reasonable request.
